# The role text structure inference skill plays for eighth graders’ expository text comprehension

**DOI:** 10.1007/s11145-017-9801-x

**Published:** 2017-11-18

**Authors:** Camille Welie, Rob Schoonen, Folkert Kuiken

**Affiliations:** 1 Amsterdam University of Applied Sciences, Amsterdam, The Netherlands; 2 Radboud University, Nijmegen, The Netherlands; 3 University of Amsterdam, Amsterdam, The Netherlands

**Keywords:** Text structure inference skill, Expository text comprehension, Secondary school, Language minority students

## Abstract

The present study investigated whether text structure inference skill (i.e., the ability to infer overall text structure) has unique predictive value for expository text comprehension on top of the variance accounted for by sentence reading fluency, linguistic knowledge and metacognitive knowledge. Furthermore, it was examined whether the unique predictive value of text structure inference skill differs between monolingual and bilingual Dutch students or students who vary in reading proficiency, reading fluency or linguistic knowledge levels. One hundred fifty-one eighth graders took tests that tapped into their expository text comprehension, sentence reading fluency, linguistic knowledge, metacognitive knowledge, and text structure inference skill. Multilevel regression analyses revealed that text structure inference skill has no unique predictive value for eighth graders’ expository text comprehension controlling for reading fluency, linguistic knowledge and metacognitive knowledge. However, text structure inference skill has unique predictive value for expository text comprehension in models that do not include both knowledge of connectives and metacognitive knowledge as control variables, stressing the importance of these two cognitions for text structure inference skill. Moreover, the predictive value of text structure inference skill does not depend on readers’ language backgrounds or on their reading proficiency, reading fluency or vocabulary knowledge levels. We conclude our paper with the limitations of our study as well as the research and practical implications.

## Introduction

Reading a text should lead to the construction of a mental representation of a text (Kintsch & Rawson, 2005; Kintsch, 1998). In Kintsch’ construction-integration model (Kintsch & Rawson, 2005; Kintsch, 1998), the final representation is called the situation model, which is a combination of text-based information and information that is integrated in the representation by knowledge-based inferences. The situation model distinguishes between two levels of representation: the local level (i.e., the word and sentence level), also known as the microstructure, and the global level (i.e., the overall or text level), also known as the macrostructure (Kintsch & Rawson, 2005; Kintsch, 1998). A reader creates coherence in the representation by inferring the type of relationship that exists between text parts, using background knowledge and/or cohesive devices (i.e., connectives, signaling words) that signal the relationship between text parts.

Besides Kintsch’ construction-integration model, several theories about reading comprehension, such as the framework for reading comprehension (Perfetti, 1999; Perfetti, Landi, & Oakhill, 2005) and the simple view of reading (Gough, Hoover, & Peterson, 1996; Gough & Tunmer, 1986; Hoover & Gough, 1990), have identified the knowledge and skills one requires to comprehend a text and to build a coherent and accurate mental representation. Vocabulary knowledge is an essential component in all these theories (Kintsch & Rawson, 2005; Perfetti et al., 2005; Hoover & Gough, 1990). A reader needs to have vocabulary knowledge to attach meaning to a word once it is decoded. Knowledge about reading strategies (metacognitive knowledge) is another type of knowledge that has been put forward as important to reading comprehension by these theories, except for the simple view of reading, which does not explicitly mentions the importance of this component (see for example Kirby & Savage, 2008). Several studies, however, have shown that metacognitive knowledge related to reading comprehension accounting for the linguistic knowledge component in the simple view of reading (e.g., Cromley & Azevedo, 2007; Schoonen, Hulstijn, & Bossers, 1998; Trapman, van Gelderen, van Steensel, van Schooten, & Hulstijn, 2014; Van Gelderen, Schoonen, Stoel, de Glopper, & Hulstijn, 2007). Metacognitive knowledge, among other things, helps readers to solve comprehension problems during reading.

Besides knowledge, fluent access to knowledge is also considered pivotal to reading comprehension. The importance of reading fluency is in particular emphasized in Perfetti’s framework for reading comprehension (Perfetti, 1999; Perfetti et al., 2005). According to this framework, fluency in lower-order processing (i.e., word and sentence processing) is a prerequisite for the successful execution of higher-order comprehension processes because of readers’ limited working memory capacity. According to Perfetti’s framework, readers do not have enough working memory capacity available for the execution of higher-order comprehension processes if lower-order processing is slow and effortful, requiring considerable capacity.

Although the abovementioned theories have identified the types of knowledge and skills one requires to comprehend a text, less is known about the knowledge and skills one requires to understand specific text genres. In the present study, we investigate the knowledge and skills readers require for understanding expository texts. The reason to focus on expository texts is that these texts have been shown to be difficult for a large proportion of secondary school readers (Hacquebord, Linthorst, Stellingwerf, & de Zeeuw, 2004; Inspectie van het onderwijs, 2008; Kamil, 2003; Lemke et al., 2004; OECD, 2003, 2007; Perie, Grigg, & Donahue, 2005; Welie, 2017). In the Netherlands, for example, between 20 and 30% of the readers have been estimated to fail the desired level of text understanding (Hacquebord et al., 2004). Furthermore, text comprehension problems have been shown especially an issue for readers with a language minority background who do not speak the majority language at home (for a review in North-American context, see August & Shanahan, 2006; for the Netherlands, see, for example Aarts & Verhoeven, 1999; Trapman et al., 2014; Van Gelderen et al., 2003).

In this context, the present study is set up to get a better understanding of the knowledge and skills important for secondary school readers’ expository text comprehension. In particular, the present study focuses on one specific skill, namely the ability to infer text structure, which we consider an especially helpful skill for expository text comprehension. Furthermore, the present study examines whether language background, reading proficiency, sentence reading fluency and linguistic knowledge might affect the contribution of text structure inference skill to text comprehension. In what follows we will first explain why we assume that the ability to infer text structure is an especially helpful skill for expository text comprehension. Next, we will argue why benefits of this skill might depend on a reader’s language background, reading proficiency, reading fluency or linguistic knowledge.

## Text structure inference in expository text comprehension

Meyer (1985) reported that the overall organization, or top-level structure, of most expository texts can be described by one of five patterns: problem–solution, causation, description, comparison and collection/sequence. If a text is organized by one of these top-level structures, we expect readers who infer this top-level structure to have a better text comprehension than readers who do not. In line with Meyer, Brandt, and Bluth (1980), we assume that a reader who infers a text’s top level structure is facilitated in building a coherent representation of a text, because inferring this structure will help the reader to hierarchically store text information. That is, once a top-level structure is inferred we assume that a reader will use this structure (or schema) as a guiding principle to distinguish between more and less important text information and to store text information accordingly; more specifically, text information linked to the text’s top level structure is stored at the highest level in the hierarchy of a text representation and details supporting the overall structure are stored at lower levels in the representation. Readers who do not infer the top-level structure of a text will not have a schema guiding them how to store hierarchically the text information they encounter; their text representation is therefore expected to be list-like, lacking hierarchical organization of ideas.

Findings from Meyer et al. (1980) support the link between text structure inference skill and text comprehension. In their study, readers who were better able to infer the top-level structure of a text also had higher text comprehension levels. Moreover, intervention studies seem to suggest a causal link between text structure inference skill and text comprehension because training students to attend to text structure during reading (e.g., underlining words that signal text structure and searching for the overall structure of a text) is associated with better scores on standardized reading comprehension tests, as well as with better recall of a text’s main ideas and of text information in general (e.g., Cook & Mayer, 1988; Gordon, 1989; Meyer, Young, & Bartlett, 1989; Meyer & Poon, 2001; Paris, Cross, & Lipson, 1984; Wijekumar, Meyer, & Lei, 2013; Williams, Hall, & Lauer, 2004; Williams, 2005; Williams, Stafford, Lauer, Hall, & Pollini, 2009).

## Characteristics that may influence text structure inference

Readers may not benefit equally from their text structure inference skills to improve their text comprehension. Four factors in particular may influence the benefits of inferring text structure: language background, reading proficiency, reading fluency and linguistic knowledge. As regards language background, Hacquebord (1989, 1999) assumed that readers with a language minority background may focus more on higher levels of text processing compared to their monolingual peers as a compensating mechanism for language problems at lower levels of text processing, i.e., at the word and sentence level. Her assumption was based on the finding that language minority readers did not perform worse than their monolingual peers on questions that tapped into global text comprehension, whereas the language minority readers were less able to answer text comprehension questions that assessed word and sentence level comprehension. From this compensatory view the relationship between text structure inference skill and reading comprehension is expected to be stronger for language minority readers than for their monolingual peers, as it is expected that language minority readers use this skill to compensate for text comprehension problems at lower text levels.

Results from Stevenson, Schoonen, and de Glopper (2003) do not support Hacquebord’s compensatory view. They found, by means of a think-aloud study, that in comparison to their monolingual peers, language minority readers used more reading strategies that focused directly on their language problems instead of compensating for these problems by focusing on higher text levels. Other studies also concur with findings of Stevenson et al. (2003): readers with problems at the word and sentence level do not seem to use a more top-down approach of text processing, but instead seem to focus directly on their problems at the word and sentence level (e.g., Davis & Bistodeau, 1993; Horiba, 1990, 1996, 2000). If language minority readers indeed direct more attention to word and sentence level processing (due to their limited reading fluency or linguistic knowledge), the attentional resources this requires may hamper them to engage in higher order processing such as text structure inference (e.g., Bernhardt & Kamil, 1995; Cummins, 1979; Just & Carpenter, 1992; LaBerge & Samuels, 1974; Perfetti, 1985; Perfetti & Lesgold, 1977; Perfetti & Hart, 2001; Segalowitz, Watson, & Segalowitz, 1995). Assuming limited attentional resources and a ‘non-compensatory’ view, the relationship between text structure inference skill and reading comprehension may be less strong for language minority readers (compared with monolinguals) because cognitive load for other text processes could prevent language minority readers from inferring text structure.

In addition, if cognitive load for other processes leads to less attention for text structure, it could be the case that besides language background, reading proficiency, reading fluency and vocabulary knowledge influence text structure inference. Readers with relatively low reading proficiency, reading fluency or vocabulary knowledge are expected to require more cognitive load for processes at the word and sentence level compared to their more proficient, fluent and knowledgeable peers and may therefore not have enough capacity free for higher order processes such as inferring text structure (cf., Bernhardt & Kamil, 1995; Cummins, 1979; Just & Carpenter, 1992; LaBerge & Samuels, 1974; Perfetti, 1985; Perfetti & Lesgold, 1977; Perfetti & Hart, 2001; Segalowitz et al., 1995). For readers with a poor reading proficiency other factors may play a role as well: researchers have indicated that understanding the relevance of strategies, the motivation to employ them and sufficient practice is also required to utilize strategies during reading (e.g., Baker, 2005; Pintrich & Zusho, 2002; Veenman, van Hout-Wolters, & Afflerbach, 2006). Poor comprehenders are expected to meet these demands to a lesser extent than their more competent peers and therefore may not direct their attention to text structure to the same extent as their better comprehending peers.

## The present study

Based on previous findings we assume that text structure inference skill is an important factor for expository text comprehension in secondary school. In the present study we aimed to examine the unique contribution of this skill to eighth graders’ expository text comprehension controlling for knowledge and skills that have been put forward by various theories as important predictors of text comprehension. Moreover, we aimed to investigate whether the unique predictive value of text structure inference skill differs between readers with different language backgrounds or between readers who vary in reading proficiency, reading fluency or vocabulary knowledge. As regards language background, we compared monolingual Dutch students and bilingual Dutch students with a language minority background. We also differentiated between bilinguals with Dutch as a dominant and those with Dutch as a non-dominant language at home as we hypothesized that these groups might differ on fluency skills and linguistic knowledge, which could have an impact on the relationship between text structure skill and reading comprehension.

To examine the additional contribution of text structure inference skill, we decided to control for reading fluency, linguistic knowledge and metacognitive knowledge. Reading fluency and linguistic knowledge were used as control variables because they have been put forward as important components of reading comprehension in Kintsch’ construction-integration model (Kintsch, 1998; Kintsch & Rawson, 2005) and Perfetti’s framework for reading comprehension (Perfetti, 1999; Perfetti et al., 2005). For practical reasons, that is, the ease of testing, we decided not to measure reading accuracy. There was also a theoretical rationale for excluding reading accuracy from our test battery: studies have found that word reading accuracy was not a significant predictor for Dutch readers who were 3 years younger (Veenendaal, Groen, & Verhoeven, 2015) and 1 year younger (Trapman et al., 2014) than our participants. Based on these results, we think that reading accuracy is not important for predicting individual differences in reading comprehension at secondary school, in particular because Dutch has a relatively high orthographic transparency (cf., Veenendaal et al,, 2015). We also excluded word reading fluency from our test battery as we assume that also fluency at the word level is not an important distinguishing feature for reading comprehension performance at secondary school. This assumption concurs with studies that have found that word recognition fluency was not related to seventh and eighth graders’ reading comprehension (Trapman et al., 2014; Van Gelderen et al., 2007), not even for the low achievers in seventh grade (Trapman et al., 2014).

We also controlled for metacognitive knowledge because several studies have shown that metacognitive knowledge and skills account for unique variance in secondary school readers’ text comprehension above and beyond linguistic knowledge (e.g., Cromley & Azevedo, 2007; Schoonen et al., 1998; Trapman et al., 2014; Van Gelderen et al., 2007). Including metacognitive knowledge as an additional control variable also made it possible to examine to what extent having knowledge about text structure (metacognitive knowledge) and the application of this knowledge (inferring text structure) relate uniquely to expository text comprehension. Our aims led to the following two research questions:

1. Does text structure inference skill contribute to eighth graders’ reading comprehension above and beyond reading fluency, linguistic knowledge and metacognitive knowledge?
2. Does the unique contribution of text structure inference skill depends on readers’ language backgrounds or their reading proficiency, reading fluency or linguistic knowledge levels?

In the next section we will discuss the method we used to answer these questions followed by the results and discussion section.

## Method^1^

### Participants

The study started with 337 students from thirteen eight grade classes (ages 13–15) from three secondary schools. Students were excluded from the analyses when they had learning or reading problems according to school reports (n = 16) or when they showed disobedient behavior on one or more class administered tests according to the test administrator’s notes (n = 92). The large attrition due to misbehavior is related to the difficult school population and teachers’ challenges in classroom management during test administration. Most attrition was on the expository text comprehension test (n = 59). Furthermore, students were also excluded when they had one or more test scores missing due to absence on a testing session or due to exclusion of their test scores (n = 38). Test scores were excluded for students who skipped half or more of the items on a test or scored below chance level, since this was regarded as an indication of test disturbance. In addition, one school decided to discontinue participation for most students after the first two testing sessions (n = 40, school B in Table 1).

**Table 1:** Students included in the analyses per school, class and the educational level of each class

School	Class	Educational level^a^	Number of students
A	A1, A2, A3, A4	Low	44
	A5	Intermediate	16
	A6, A7	High	45
		Total school A	105
B	B1	Low	7
	B2	Intermediate	3
	B3	High	6
		Total school B	16
C	C1, C2	Intermediate	14
	C3	High	16
		Total school C	30
		Total all schools	151

^a^The educational levels correspond to the following educational levels in Dutch secondary school: low = *vmbo*-*t* (prevocational level) or *vmbo*-*t/havo* (prevocational/general secondary educational level), intermediate = *havo* (general secondary educational level) or *havo/vwo* (general secondary educational/pre-university level), high = *vwo* (pre-university level)

Due to exclusion of test scores, only 191 students had valid expository text comprehension scores. We performed our analyses with a sample of 151 students without missing scores on the other tests as well. In our final sample, students received instruction at various educational levels: 34% of the students received instruction at a low educational level (n = 51), 22% of the students at an intermediate educational level (n = 33) and 44% of the students at a high educational level (n = 67). Table 1 shows the number of students per school, per class and the educational level of each class.

Students were regarded as monolingual Dutch (n = 53) when they indicated in the background questionnaire (see instruments section) that Dutch was their only mother tongue, and as bilingual Dutch (n = 98) when one or more other languages than Dutch were involved in their initial language acquisition. All but seven of the bilingual students were born in the Netherlands and only two of them had received less than 5 years primary education in the Netherlands. Bilinguals were assigned to the *Bilinguals Dutch dominant* at home group (n = 36) when they indicated that their parents spoke at least 50% of the time Dutch to them, the other bilinguals were assigned to the *Bilinguals Dutch not dominant group* (n = 62).

### Instruments

The students were submitted to six tests which measured their expository text comprehension, vocabulary knowledge (two tests), metacognitive knowledge, reading fluency and their text structure inference skill. Students also filled out a questionnaire tapping into background information. Table 2 shows the internal consistency (Cronbach’s Alpha) as reliability estimates of the tests for each subgroup. In general, the tests show satisfactory reliability estimates between .70 and .97 except for the metacognitive knowledge test and the text structure inference skill test for which reliability estimates are between .60 and .65, and .59 and .73 respectively.

**Table 2:** Reliability estimates (Cronbach’s Alpha) of the tests for the whole sample and the subsamples

	Number of items	All students (n = 151)	Monolingual Dutch (n = 53)	Bilingual Dutch (n = 98)	Bilingual Dutch dominant (n = 36)	Bilingual Dutch not dominant (n = 62)
Expository text comprehension	35	.79	.85	.72	.74	.70
General vocabulary knowledge	70	.81	.76	.79	.81	.78
Knowledge of connectives	43	.83	.86	.78	.73	.81
Metacognitive knowledge	45	.64	.60	.64	.62	.65
Sentence reading fluency (*RT in msec*)	46	.96	.96	.96	.95	.96
Text structure inference skill	30	.67	.73	.64	.70	.59

#### Expository text comprehension

The reading comprehension test comprised 35 multiple choice questions (with three or four answer options) about five expository texts. Readers had to answer questions about local and global comprehension of the texts. In order to answer these questions, readers had to infer various types of relationships between text parts (e.g., causal, contrastive), as well as infer the main idea (overall structure) of the texts. The first text about energy systems in the body had a comparison overall structure. The second text, in which the history of whaling was described, had a sequence overall structure. The third (about athletics), the fourth (about the way muscles work) and the fifth text (about sustainable house construction) were mainly descriptive, but contained elements of other structures as well on the paragraph level. In the fourth text, for example, one of the four paragraphs compared three types of muscle contraction and in the fifth text two types of house construction were compared whereas the last two paragraphs of this fifth text could be classified as describing a problem (not enough sustainable house construction) and a solution for this problem (informing constructors about the benefits of sustainable house construction). Texts varied in length between 184 and 449 words. The Hazenberg and Hulstijn (1996) list of word frequency in written Dutch was used to assess word frequency of the words in the texts: 88% of the words in the texts belonged to the 2000 most frequent words in the list and 5% of the words belonged to the 2000–5000 most frequent words in the list. Four texts were derived from the database of *Diataal*, a Dutch test institute (Hacquebord, Stellingwerf, Linthorst, & Andringa, 2005). One text was derived from the reading comprehension test used in a study by Van Gelderen et al. (2007). Texts and questions were slightly adapted.

#### Linguistic knowledge

Two tests measured linguistic knowledge. One was a digitally administered *general vocabulary knowledge* test developed by *Diataal* (Hacquebord et al., 2005) which included 70 multiple choice items drawn from a corpus of school book texts. Test items varied in difficulty level (as judged by teachers) and frequency in the corpus. Items were general academic words, for example ‘aspects’, as well as domain or subject specific words, for example ‘roam’ (e.g., in a forest), ‘interior’ (i.e., of a house) or ‘executed’ (i.e., murdered).

The other vocabulary knowledge test tapped into students’ *knowledge of connectives* by means of a 43 item fill-in-the-blanks test consisting of six short expository texts which varied in length between 85 and 177 words and which addressed various topics (e.g., spiders, vitamins, the origin of the @-symbol, etc.). For each blank, students had to choose the appropriate connective out of three options. Relationships between the propositions that had to be connected were regarded as familiar to all students. To ensure that the texts did not posit any other vocabulary knowledge demands on the selection of the right connective (i.e. other than knowledge of connectives), texts contained for 95% words (or transparent derivations/inflections of these words) that belong to the 5000 most frequent words in written Dutch according to the Hazenberg and Hulstijn (1996) list (85% 0–2000; 10% 2000–5000). The remaining 5% of the words (predominantly proper names) were not in the Hazenberg and Hulstijn list but were considered not to cause any difficulties for selecting the right connective.

The knowledge of 43 connectives from various semantic classes was tested. Connectives expressed *additive*-*positive* (7), *additive*-*negative* (6, also known as *contrastive*), *temporal* (8), *causal* (10) and *adversative* (4) relationships (e.g., Crosson, Lesaux, & Martiniello, 2008; Halliday & Hasan, 1976; Sanders & Spooren, 2007; Sanders, Spooren, & Noordman, 1992). In addition, in accordance with McNamara, Graesser, and Louwerse (2012), the test contained *additive*-*clarifying* (8) connectives. Connectives varied in difficulty level and were matched with distractors of corresponding difficulty levels in order to reduce the possibility that test takers could benefit from their knowledge of relatively easy distractors in their selection of the target connective. Distractors were chosen that could fit the blank syntactically, but only the targets fitted the blank semantically. Five expert readers (researchers) had 100% agreement on the correct responses. To determine the difficulty level of connectives and distractors, results from Hacquebord, Alberts, and Andringa (2011) were used. In that study, 68 secondary school teachers were asked to rate the expected difficulty of words from school book texts on a scale from one (very easy, known at the end of primary school) to five (too hard and/or irrelevant, not known at the end of eight grade); for each word the mean difficulty level was computed. The test contained 22 connectives with a low (mean judgment from 1 to 2.3), 16 with a medium (mean judgment from 2.4 to 3.6) and 5 with a high difficulty level (mean judgment from 3.6 to 5). Each of the six semantic classes contained connectives from at least two difficulty levels. Most distractors differed between 0 and 1.3 points (within the range of a difficulty level) in difficulty from the target items, except for eight distractors which differed from 1.4 to 2 points in difficulty from the target.

#### Metacognitive knowledge

To measure students’ metacognitive knowledge of text structure and reading and writing strategies, we used an adapted version of the metacognitive knowledge test used by Van Gelderen et al. (2007). The original test was reduced to 45 statements. In this test participants had to indicate whether or not they agreed with statements about text structure and writing and reading strategies. For example, a correct response would be if they agreed with the following statement: ‘if you do not understand the meaning of a word, it is useful to try and guess its meaning by looking at other words and sentences surrounding the unknown word’.

#### Sentence reading fluency

Sentence reading fluency was measured by a sentence verification test similar to the one used by Van Gelderen et al. (2007). Students were presented 110 sentences on a laptop screen and had to decide as fast as possible whether a sentence made sense or not by pressing a red (sentence makes no sense) or a green stickered key (sentence makes sense) on their laptops’ keyboards. Half of the sentences made sense, the others did not. Sentences that did not make sense were in flagrant contradiction with encyclopedic knowledge all students were considered to share (e.g., ‘Most bicycles have seven wheels’ and ‘The Netherlands is the biggest country in the world’ were sentences that did not make sense). Reading fluency was calculated by averaging the reaction times on the correct responses to the sentences that make sense.

#### Text structure inference skill

Before students started with the text structure inference skill test they received an oral instruction by a trained test-assistant. This instruction was also printed in their booklets, but because some students might skip this instruction it was provided orally as well to ascertain that students knew what was expected from them. Students had to indicate the ‘main structure’ (i.e., top-level structure) of short texts by means of answering a multiple choice question and they had to summarize these texts in no more than two sentences. The ‘main structure’ of the text was explained as follows (translated from Dutch):

The main structure of a text is the most important structure of a text, the way in which a text is organized. You will read a short text. Afterwards you will have to indicate what the main structure of the text is. You can choose from the following options:

The text:

- describes a cause and one or more consequences
- describes a problem and one or more solutions
- gives more information about a subject
- gives more information about a subject in a certain order (e.g., sequence in time)
- compares matters with each other

Note that in this test you have to indicate *the most important structure* of the text. It may be that there are also other relationships between sentences in the text; for example, a text could have a main structure problem–solution but matters in the text may be compared as well.

Most important in this test is thus the general organization of the text, the main structure.

Students were also told that they had to write a summary of no more than two sentences after they had chosen the ‘main structure’ of the text out of the five options mentioned in the example above. Next they were provided with an example of a short text and an appropriate summary for this text. As a wrap up of the instruction they were told that the test comprised three steps: (1) reading the text, (2) indicating the ‘main structure’ of the text and (3) summarizing the text in no more than two sentences. It was stressed that only one answer had to be chosen in the multiple choice question about the main structure of the text.

The test consisted of 15 short expository texts of one or two paragraphs. The texts varied in length from 78 to 244 words (the average text length was 110 words) and addressed various topics (animals, boats, history of the car, obesity, etc.). Texts were organized with one of the five basic patterns of expository texts identified by Meyer (1985). Four texts had a cause-consequence structure, three a problem–solution, three were descriptions (more information about a topic), two were descriptions in a certain sequence and three texts made comparisons between matters.

The summaries students wrote about the texts were scored by two independent raters. Summaries could be awarded 0, 1, 1.5 or 2 points. Zero points were awarded when the top-level structure was not present in the summary. Two points were allocated when the main structure was present: for main structures comprising two parts (cause-consequence, problem–solution, comparison of two things) both parts had to be present; for summaries from the texts with a description or sequence top-level structure two points were awarded when it was clear from the summary that the appropriate text structure was inferred. Summaries from causation, problem–solution or comparison texts were awarded one point when the summary comprised one of the two parts of the main structure (e.g., only the problem in a problem–solution text). Two texts could be awarded 1.5 points. One text had a problem–solution top-level structure with two solutions; if the problem and only one of the solutions was mentioned in the summary 1.5 points were awarded. One texts had a cause-consequence structure with three consequences; if the cause and only one consequence was mentioned in the summary 1.5 points were allocated (none of the summaries mentioned the cause and two consequences for this text). The average score of the two raters was used for further analysis. Rater reliability was computed as Intra Class Correlation (ICC) and turned out to be .97. The total score on the test, with a maximum of 45, was computed by adding up the correct scores on the multiple choice questions (one point per correct answer) and the scores on the summaries.

#### Background questionnaire

The background questionnaire asked for the following information: gender, country of birth, mother tongue, language(s) the parents/caretakers speak to participants (and percentages of the time they speak these languages to them), country of birth of parents/caretakers, the highest completed educational level of parents/caretakers and jobs of parents/caretakers.

### Procedure

From March till June 2014 tests were administered, each in a separate testing session. Students had enough time to complete the tests. All tests were administered during regular classes except for the sentence reading fluency test for which participants in groups of four were taken out of their regular classes to a separate test room. Test administrators took notes of students’ behavior during plenary test administrations.

### Scoring and missing value treatment

On the general vocabulary knowledge and the sentence reading fluency test there were no missing responses because these digital tests required a response on every item. Incidentally skipped items from the expository text comprehension, knowledge of connectives, metacognitive knowledge and the text structure inference skill test were scored as incorrect. For the sentence reading fluency test the procedure described in Van Gelderen et al. (2003) was used for scoring and missing value treatment. First, to ensure that linguistic knowledge and comprehension did not influence performance on the fluency test, sentences with a lower accuracy rate than .875 (i.e., in accordance with Van Gelderen et al., 2003) were excluded from the analysis. Nine sentences in the reading fluency test were deleted (hence mean reaction times were calculated on the remaining 46 sentences). Second, inaccurate responses to sentences or potentially untrustworthy ones (extremely slow responses, i.e., three standard deviations above the mean, or extremely fast responses, i.e., faster than the fastest reaction time of a group of five expert readers) were turned into missing values. Next, the missing values on the sentences in the reading fluency test were estimated with the expectation maximization procedure of SPSS. After this procedure, the mean reaction time for the sentence reading fluency test was calculated per participant.

### Analyses

Means and standard deviations on all tests were computed for the whole sample and separately for the monolingual and the two bilingual subgroups (Dutch dominant versus Dutch not dominant). Because students came from different classes all regression analyses were performed with a random intercept for class (i.e., multilevel regression analyses with the formula *y*_*ij* =_ *β*_0_ + *β*_1_ *x*_*ij*_ + *u*_*j*_ + *e*_*ij*_). Differences between monolinguals and bilingual Dutch students and between the two bilingual subgroups on the tests were investigated by the use of hierarchical regression analyses with the tests as dependent variables and two independent (i.e., orthogonal) contrasts as predictor variables: one predictor contrasting monolingual versus bilingual Dutch students and one contrasting the two bilingual groups. These contrasts were added as predictors of the test concerned; first it was examined whether monolinguals differed from bilinguals on a test, next potential differences between the two bilingual groups were examined. Effect sizes of the differences are reported as the percentage of explained variance (Δr^2^). Furthermore, for the whole sample and for the various subsamples correlations between the test scores were calculated.

Before we investigated our research questions we investigated for each of the predictor variables (i.e., sentence reading fluency, general vocabulary knowledge, knowledge of connectives, metacognitive knowledge and text structure inference skill) whether they were related curvilinearly to text comprehension, because it has been shown that curvilinear relationships between predictors and dependent variables may affect the estimation of interaction effects (Ganzach, 1997). We investigated this by examining whether the quadratic terms of the predictors led to model improvement for each of the predictors separately.

To answer whether text structure inference skill contributed uniquely to text comprehension (our first research question) we performed a hierarchical regression analysis with text comprehension as a dependent variable. As a first step reading fluency, vocabulary knowledge (general vocabulary knowledge and knowledge of connectives) and metacognitive knowledge were added as predictors of text comprehension and as a second step text structure inference skill was added as an additional predictor to examine whether text comprehension was better accounted for with a model that also includes text structure inference skill.

To examine whether the additional contribution of text structure inference skill to text comprehension differs between monolingual and bilingual students and between readers who vary in reading proficiency, reading fluency and vocabulary knowledge levels (our second research question) we tested whether including an interaction of text structure inference skill with one of the potential moderators (language background, reading proficiency, etc.) predicted text comprehension better than a model without this interaction. The interaction between text structure inference skill and reading proficiency level was tested by means of two dummy variables that differentiated between the 50% best scoring (n = 76) and the 50% worst scoring (n = 75) students on the text comprehension test; good comprehenders had a score of 1 and poor comprehenders a score of 0 on the variable ‘dummy good’ and scoring was vice versa for the variable ‘dummy poor’. These two dummy variables were entered as predictors of text comprehension along with text structure inference skill, reading fluency, vocabulary knowledge and metacognitive knowledge. As a second step the interaction between text structure inference skill and ‘dummy poor’ was entered to investigate if poor and good comprehenders differ significantly from each other in the relationship between text structure inference skill and text comprehension (see for a similar method Rijkeboer, van den Bergh, & van den Bout, 2011).

We also performed the abovementioned regression analyses with a sample size of 191 students to check for the robustness of our results. These 191 students all had a score on expository text comprehension and 40 students had a score missing on one (n = 32), two (n = 7) or three (n = 1) of the predictor variables. For our regression analyses with this sample we created dummy variable for each predictor which represented whether a score was missing (a score of 1) or not (a score of 0) on the associated predictor. We entered these dummy variables along with the associated predictor variables in our regression models. These regression models did not include a fixed intercept and missing scores on the standardized predictor variables were recoded into a score of zero (see Koomen & Hoeksma, 1991). This method enabled us to investigate if the outcomes of our models were different than with the sample of 151 students when our models controlled for the variance that was accounted for in text comprehension by differences between students who either missed or did not miss a score on each of the predictor variables.

## Results

### Descriptive statistics

Expository text comprehension scores were normalized with Blom’s formula (Blom, 1958). Table 3 shows the means and standard deviations on the six tests for the whole sample and for the various subgroups. Regression analyses indicated that the monolinguals outperformed the bilinguals in expository text comprehension (*χ* ^2^ (1) = 6.07, *p* = .01, Δr^2^ = .06), general vocabulary knowledge (*χ* ^2^ (1) = 15.56, *p* = .00, Δr^2^ = .14), knowledge of connectives (*χ* ^2^ (1) = 9.98, *p* = .00, Δr^2^ = .09) and metacognitive knowledge (*χ* ^2^ (1) = 4.03, *p* = .04, Δr^2^ = .05), but not in sentence reading fluency (*χ* ^2^ (1) = .01, *p* = .92, Δr^2^ = .00) and text structure inference skill (*χ* ^2^ (1) = .35, *p* = .55, Δr^2^ = .00). The bilingual Dutch dominant group outperformed the bilingual Dutch not dominant group in sentence reading fluency (*χ* ^2^ (1) = 7.44, *p* = .01, Δr^2^ = .04), but there were no differences between the two bilingual groups on expository text comprehension (*χ* ^2^ (1) = 1.10, *p* = .29, Δr^2^ = .01), knowledge of connectives (*χ* ^2^ (1) = .26, *p* = .61, Δr^2^ = .00), general vocabulary knowledge (*χ* ^2^ (1) = .80, *p* = .37, Δr^2^ = .00), metacognitive knowledge (*χ* ^2^ (1) = 2.41, *p* = .12, Δr^2^ = .01) and text structure inference skill (*χ* ^2^ (1) = .22, *p* = .64, Δr^2^ = .00).

**Table 3:** Means (and standard deviations) on the six measures for the whole sample and the subgroups

	Number of items	All students (n = 151)	Monolingual Dutch (n = 53)	Bilingual Dutch (n = 98)	Bilingual Dutch dominant (n = 36)	Bilingual Dutch not dominant (n = 62)
Expository text comprehension	35	25.11 (5.22)	26.78 (5.98)	24.20 (4.54)	23.53 (4.84)	24.59 (4.35)
General vocabulary knowledge	70	53.24 (7.51)	57.04 (6.17)	51.18 (7.40)	51.89 (7.68)	50.77 (7.26)
Knowledge of connectives	43	31.89 (5.85)	34.34 (5.88)	30.57 (5.42)	30.33 (4.84)	30.71 (5.77)
Metacognitive knowledge	45	35.79 (4.08)	37.00 (3.64)	35.13 (4.16)	34.39 (4.20)	35.56 (4.11)
Reading fluency (*RT in msec*)	46	2828 (500)	2816 (504)	2835 (500)	2659 (459)	2937 (498)
Text structure inference skill	30 (max. score 45)	26.02 (5.64)	25.81 (5.98)	26.14 (5.47)	25.69 (6.05)	26.40 (5.13)

### Correlations

Table 4 shows the correlations between the six variables for the whole sample and for the subgroups. The knowledge variables general vocabulary knowledge, knowledge of connectives and metacognitive knowledge correlated positively with reading comprehension: correlations were low to moderate (between .32 and .65). Positive correlations between reading comprehension and text structure inference skill were also low to moderate (between .28 and .60). Reading comprehension correlated weakly with sentence reading fluency (correlations between − .10 and − .17). Correlations between text structure inference skill and the knowledge variables ranged from low to strong (correlations between .16 to .71); between text structure inference skill and reading fluency correlations were low (between − .11 and − .39). The correlations between the knowledge variables ranged from low to high (from .29 to .74) and reading fluency correlated low to moderate with the knowledge variables (correlations from − .19 to − .44).

**Table 4:** Correlations between the six variables for the whole sample and the various subgroups

	General vocabulary knowledge	Knowledge of connectives	Metacognitive knowledge	Sentence reading fluency	Text structure inference skill
*Expository text comprehension*					
All students	.44*	.56*	.47*	− .12	.38*
MD	.38*	.55*	.51*	− .15	.36*
BD	.41*	.52*	.42*	− .10	.42*
BDdom	.56*	.65*	.55*	− .17	.60*
BDndom	.32*	.47*	.32*	− .12	.28*
*General vocabulary knowledge*					
All students		.52*	.45*	− .33*	.37*
MD		.51*	.31*	− .44*	.47*
BD		.44*	.45*	− .31*	.38*
BDdom		.65*	.74*	− .40*	.71*
BDndom		.34*	.29*	− .24	.16
*Knowledge of connectives*					
All students			.44*	− .29*	.38*
MD			.44*	− .42*	.53*
BD			.38*	− .23*	.33*
BDdom			.51*	− .32	.38*
BDndom			.32*	− .22	.31*
*Metacognitive knowledge*					
All students				− .22*	.36*
MD				− .19	.42*
BD				− .23*	.35*
BDdom				− .40*	.55*
BDndom				− .22	.21
*Sentence reading fluency*					
All students					− .22*
MD					− .26
BD					− .19
BDdom					− .39*
BDndom					− .11

* *p* < .05

*MD* monolingual Dutch (n = 53), *BD* bilingual Dutch (n = 98), *BDdom* bilingual Dutch dominant (n = 36), *BDndom* bilingual Dutch not dominant (n = 62)

### Curvilinear effects

We could not establish a curvilinear relationship with text comprehension for sentence reading fluency (*χ* ^2^ (1) = .00, *p* = 1.00, Δr^2^ = .00), general vocabulary knowledge (*χ* ^2^ (1) = 3.17, *p* = .08, Δr^2^ = .02), metacognitive knowledge (*χ* ^2^ (1) = 1.95, *p* = .16, Δr^2^ = .00) and text structure inference skill (*χ* ^2^ (1) = .70, *p* = .40, Δr^2^ = .00). For knowledge of connectives the quadratic term did lead to model improvement on top of the linear term (*χ* ^2^ (1) = 3.97, *p* = .04, Δr^2^ = .01) but we considered the curvilinear relationship invalid because adding the quadratic term led to non-significance of the linear term (cf., Breetvelt, Van den Bergh, & Rijlaarsdam, 1994).

### Effects of text structure inference skill (research question 1)

Table 5 shows the results of the regression analyses to answer our research questions. This table demonstrates that adding text structure inference skill as a predictor of text comprehension in addition to sentence reading fluency, general vocabulary knowledge, knowledge of connectives and metacognitive knowledges does not improve model fit significantly, compare model 2 versus model 1, *χ* ^2^ (1) = 2.79, *p* = .09, Δr^2^ = .01.

**Table 5:** Model fit, variance components and parameter estimates for our two research questions (N_students_ = 151, N_classes_ = 13)

Research questions	RQ 1			RQ 2						
Models	M0	M1	M2	M3	M4	M5	M6	M7	M8	M9
Variance										
Class	.18 (.10)	.03 (.03)	.03 (.03)	.03 (.03)	.03 (.03)	.01 (.01)	.01 (.01)	.03 (.03)	.03 (.03)	.02 (.09)
Students	.79 (.09)	.56 (.07)	.55 (.07)	.55 (.07)	.55 (.07)	.29 (.04)	.29 (.03)	.55 (.07)	.56 (.07)	.55 (.09)
Total	.97	.59	.58	.58	.58	.30	.30	.58	.59	.57
Distribution of variance										
Class	18.6%	5.0%	5.2%	5.2%	5.2%	3.3%	3.3%	5.2%	5.1%	3.5%
Students	81.4%	95%	94.8%	94.8%	94.8%	96.7%	96.7%	94.8%	94.9%	96.5%
Explained variance										
Class		83.3%	83.3%	83.3%	83.3%	94.4%	94.4%	83.3%	83.3%	88.8%
Students		29.1%	30.4%	30.4%	30.4%	63.3%	63.3%	30.4%	29.1%	30.4%
Total		39.2%	40.2%	40.2%	40.2%	69.1%	69.1%	40.2%	39.2%	41.2%
Increase in explained variance										
Class		83.3%	–	–	–	11.1%	–	–	–	5.5%
Students		29.1%	1.3%	–	–	32.9%	–	–	–	–
Total		39.2%	1.0%	–	–	28.9%	–	–	–	1.0%
Fit in − 2LL	408.12	348.30	345.51	344.97	343.94	246.15	244.85	344.90	345.26	344.27
Difference in − 2LL		59.82*	2.79	.54	1.03	97.79*	1.30	.61	.25	1.24
Difference in *df* Compared to model		4 M0	1 M1	2 M2	2 M3	1 M2	1 M5	1 M2	1 M2	1 M2

Research questions	RQ 1			RQ 2						
Parameter estimates	M0	M1	M2	M3	M4	M5	M6	M7	M8	M9
Intercept	−.11 (.14)	−.02 (.08)	−.01 (.08)	−.02 (.08)	−.02 (.08)	−.67* (.08)	−.65* (.08)	−.00 (.08)	−.02 (.08)	−.03 (.08)
Sentence reading fluency		.11 (.07)	.11 (.07)	.10 (.07)	.10 (.07)	.03 (.05)	.04 (.05)	.12 (.07)	.12* (.07)	.10 (.07)
General vocabulary knowledge		.13 (.08)	.11 (.08)	.10 (.08)	.08 (.08)	.08 (.06)	.08 (.06)	.13 (.08)	.11 (.08)	.10 (.08)
Knowledge of connectives		.40* (.08)	.37* (.08)	.36* (.08)	.37* (.08)	.08 (.06)	.09 (.06)	.36* (.08)	.37* (.08)	.38* (.08)
Metacognitive knowledge		.25* (.07)	.22* (.07)	.22* (.07)	.21* (.07)	.09 (.05)	.09 (.05)	.22* (.07)	.22* (.07)	.22* (.07)
Text structure inference skill			.12 (.07)	.13 (.07)	.14 (.07)	.09 (.05)	.03 (.07)	.12 (.07)	.12 (.07)	.13 (.07)
Language background MD versus BD (LB1)				−.03 (.05)	−.04 (.05)	–	–	–	–	–
Language background BDdom versus BDndom (LB2)				−.03 (.08)	−.02 (.08)	–	–	–	–	–
Text structure inference skill × LB1					.02 (.04)	–	–	–	–	–
Text structure inference skill × LB2					.08 (.08)	–	–	–	–	–
Dummy good						1.33* (.11)	1.32* (.11)	–	–	–
Dummy poor						0^a^ (0)	0^a^ (0)	–	–	–
Dummy poor × text structure inference skill							.11 (.09)	–	–	–
Text structure inference skill × sentence reading fluency								.06 (.08)	–	–
Text structure inference skill × general vocabulary knowledge									.03 (.06)	–
Text structure inference skill × knowledge of connectives										.06 (.05)

*LB* language background, *MD* monolingual Dutch, *BD* bilingual Dutch, *BDdom* bilingual Dutch dominant at home, *BDndom* bilingual Dutch not dominant at home

* *p* < .05

^a^This parameter is set to zero because it is redundant. The difference in − 2 Log Likelihood is Chi square distributed. Predictors and dependent variable are standardized. Standard errors between brackets

As we found that text structure inference skill correlated significantly with expository text comprehension (see Table 4), it must be one or more of the control variables that did have an impact on the relationship between text structure inference skill and expository text comprehension. Additional regression analyses were performed to clarify this issue. These regression analyses included sentence reading fluency (non-significant) as a predictor and combinations of the other predictors. The analyses revealed that knowledge of connectives and metacognitive knowledge were the crucial factors: on top of these two factors text structure inference skill was not a significant predictor of expository text comprehension (*χ* ^2^ (1) = 3.37, *p* = .06, Δr^2^ = .01), whereas text structure inference skill did predict expository text comprehension uniquely controlling for knowledge of connectives (*χ* ^2^ (1) = 6.75, *p* = .01, Δr^2^ = .04), metacognitive knowledge (*χ* ^2^ (1) = 8.01, *p* = .00, Δr^2^ = .04), both general vocabulary knowledge and metacognitive knowledge (*χ* ^2^ (1) = 5.56, *p* = .02, Δr^2^ = .02), and both general vocabulary knowledge and knowledge of connectives (*χ* ^2^ (1) = 5.06, *p* = .02, Δr^2^ = .02).

### Interactions with text structure inference skill (research question 2)

Table 5 also demonstrates that the effect of text structure inference skill (controlling for sentence reading fluency, general vocabulary knowledge, knowledge of connectives and metacognitive knowledge) did not differ between monolinguals and bilinguals, between the two bilingual groups and between the poor and good comprehenders. Compare model 4 with model 3 for the examination of the interaction between text structure inference skill and language background (*χ* ^2^ (2) = 1.03, *p* = .31, Δr^2^ = .00) and model 6 with model 5 for the examination of the interaction between text structure inference skill and reading proficiency level (*χ* ^2^ (1) = 1.30, *p* = .25, Δr^2^ = .00). Also for readers who vary in sentence reading fluency, general vocabulary knowledge and knowledge of connectives levels the unique contribution of text structure inference skill to text comprehension did not differ significantly. Compare in Table 5 model 7 with model 2 for the interaction of text structure inference skill with sentence reading fluency (*χ* ^2^ (1) = .61, *p* = .43, Δr^2^ = .00), model 8 with model 2 for the interaction with general vocabulary knowledge (*χ* ^2^ (1) = .25, *p* = .62, Δr^2^ = .00) and model 9 with model 2 for the interaction with knowledge of connectives (*χ* ^2^ (1) = 1.24, *p* = .27, Δr^2^ = .01).

### Robustness check: models with 191 students

Regression analyses performed with a sample of 191 students revealed that there were no differences between expository text comprehension scores of students who either missed or did not miss a score on sentence reading fluency (*t* (191) = 1.66, *p* = .10), general vocabulary knowledge (*t* (191) = .66, *p* = .51) and knowledge of connectives (*t* (191) = − 1.16, *p* = .25). However, students who missed a score on metacognitive knowledge or text structure inference skill performed lower on expository text comprehension than those students with valid scores on metacognitive knowledge (*t* (191) = − 2.87, *p* = .01) and text structure inference skill (*t* (191) = − 3.49, *p* = .00). The results of models with a sample of 191 students were mostly similar to those with a sample of 151 students. One discrepancy was that text structure inference skill accounted for unique variance in addition to the control variables with a sample of 191 students (*χ* ^2^ (2) = 13.73, *p* = .00, Δr^2^ = .05), whereas this was not the case with the sample of 151 students (see section ‘effects of text structure inference skill’). However, this discrepancy was small; the standardized parameter estimate of text structure inference skill in the model with 151 students was .12 (standard error .07) and its *p* value was .08, whereas the standardized parameter estimate of text structure inference skill in the model with 191 students was .13 (standard error .06) and its *p* value was just below significance level, *p* = .044.

Similar to the models with 151 students, in our models with a sample size of 191 students, text structure inference skill accounted for more unique variance when our models did not include both metacognitive knowledge and knowledge of connectives as predictors, stressing the importance of these two factors for text structure inference skill. More precisely, in a model that included both metacognitive knowledge and knowledge of connectives as predictors, text structure inference skill accounted for 5% unique variance in expository text comprehension (*χ* ^2^ (2) = 13.49, *p* = .00, Δr^2^ = .05), whereas its unique variance was between 6 and 9% in regression models that did not include both these predictors.^2^ Furthermore, similar to the models with 151 students, in models with 191 students, the effect of text structure inference skill was also not moderated by language background (*χ* ^2^ (2) = .66, *p* = .42, Δr^2^ = .00), reading proficiency (*χ* ^2^ (1) = 1.01, *p* = .31, Δr^2^ = .00), sentence reading fluency (*χ* ^2^ (1) = .39, *p* = .53, Δr^2^ = .00), general vocabulary knowledge (*χ* ^2^ (1) = .49, *p* = .48, Δr^2^ = .00) or knowledge of connectives (*χ* ^2^ (1) = 1.46, *p* = .23, Δr^2^ = .01).

## Discussion

The present study examined whether text structure inference skill, i.e., the ability to infer the overall structure of a text, predicts eighth graders’ expository text comprehension on top of the variance accounted for by sentence reading fluency, linguistic knowledge and metacognitive knowledge. Moreover, it was examined whether the predictive value of text structure inference skill for expository text comprehension differs between monolingual and bilingual Dutch students or between readers who vary in reading proficiency, reading fluency or linguistic knowledge levels. We found that text structure inference skill has no unique predictive value for eighth graders’ expository text comprehension. Moreover, our findings revealed that the predictive value of text structure inference skill does not differ significantly between monolingual and bilingual readers or between readers who vary in reading proficiency, sentence reading fluency or linguistic knowledge levels.

As we found that text structure inference skill correlated with expository text comprehension, we investigated when text structure inference skill predicted expository text comprehension and when it did not by means of regression analyses with several combinations of control variables. We found that text structure inference skill had no unique predictive value with both metacognitive knowledge and knowledge of connectives as control variables, whereas text structure inference skill did predict expository text comprehension with only knowledge of connectives or only metacognitive knowledge as a control variable, or when accounting for general vocabulary knowledge in addition to knowledge of connectives or metacognitive knowledge.

The finding that differences in text structure inference skill are not associated with differences in expository text comprehension when accounting for knowledge of connectives and metacognitive knowledge seems to indicate that readers who are equal in metacognitive knowledge and knowledge of connectives do not seem to differ in text structure inference skill to the extent that it results in differences in inferring text structure during expository text reading, and consequently, differences in text comprehension levels. More specifically, we argue that readers with low metacognitive knowledge and knowledge of connectives levels have too low text structure inference skills to infer text structure during expository text reading, whereas the opposite is assumed to be the case for readers with high metacognitive knowledge and knowledge of connectives levels.

This assumption is in line with Kintsch’ construction integration model (Kintsch & Rawson, 2005; Kintsch, 1998): in order to create coherence in a text, a reader needs to make use of his knowledge of connectives and metacognitive knowledge if his background knowledge is insufficient to do so. The lack of sufficient background knowledge, and hence the necessity of knowledge of connectives and metacognitive knowledge, is often clearly the case in expository texts intended to convey new information and relationships. Several studies have shown that in expository texts readers need to be informed about the way ideas are related by signaling words in order to create coherence (Degand, Lefèvre, & Bestgen, 1999; Degand & Sanders, 2002; Singer & O’Connell, 2003; Van Silfhout, Evers-Vermeul, Mak, & Sanders, 2014). It does not come as a surprise then that the knowledge enabling the use of these signaling words (i.e., knowledge of connectives and metacognitive knowledge) is crucial to infer text structure in reading expository texts. Also Meyer and colleagues have argued consistently that metacognitive knowledge and knowledge of connectives are crucial factors for inferring text structure (e.g., Meyer et al., 1980; Meyer & Rice, 1982). According to them good readers use a reading strategy—the ‘structure strategy’—to actively search for signaling words in the text and these good readers use their knowledge about signaling words and text structure to infer the overall structure of a text. What the present study underlines additionally, is that general vocabulary knowledge does not play a crucial role for inferring text structure; that is, readers with equal general vocabulary knowledge levels appear to differ substantially in their text structure inference skills, and these differences are related to text comprehension levels.

We have argued that—given a limited working memory capacity—readers with low reading fluency, linguistic knowledge or reading proficiency levels may be hampered to use their text structure inference skills as these readers’ attention may be required for the execution of other reading processes (cf., Bernhardt & Kamil, 1995; Cummins, 1979; Just & Carpenter, 1992; LaBerge & Samuels, 1974; Perfetti, 1985; Perfetti & Lesgold, 1977; Perfetti & Hart, 2001; Segalowitz et al., 1995). The lack of interaction effects, however, indicates that the relationship between text structure inference skill and expository text comprehension was not significantly weaker for readers with lower sentence reading fluency, linguistic knowledge and reading proficiency levels. It seems that these readers were not blocked more than their more knowledgeable and fluent peers to infer text structure during reading (otherwise lower correlations would have been expected). The lack of interaction between reading fluency and text structure inference skill was expected in the present study because we found no relationship between reading fluency and expository text comprehension. This finding concurs with the general developmental trend that fluency becomes less predictive of text comprehension as age increases, whereas knowledge components become more related to text comprehension as reading experience increases (see for example Hoover & Gough, 1990; Tilstra et al., 2009; Yovanoff, Duesbery, Alonzo, & Tindal, 2005). Reading fluency of eighth graders seems to have reached a level beyond which individual differences matter less for text comprehension performance.

Lastly, no interaction between language background and text structure inference skill was found. In our introductory section we hypothesized that for bilinguals with a language minority background, in comparison to their monolingual peers, the relationship between text structure inference skills and expository text comprehension might be either weaker, from a limited working memory capacity point of view (cf., Bernhardt & Kamil, 1995; Cummins, 1979; Just & Carpenter, 1992; LaBerge & Samuels, 1974; Perfetti, 1985; Perfetti & Lesgold, 1977; Perfetti & Hart, 2001; Segalowitz et al., 1995), or stronger, from a compensatory perspective. The latter perspective, advanced by Hacquebord (1989, 1999), assumes that readers with a language minority background focus more than their monolingual peers on higher levels of text processing as a compensating mechanism for language problems at the word and sentence level of text processing. Because no interaction between text structure inference skill and language background was established, we can conclude that the bilinguals in our sample did neither benefit more, nor less, from their text structure inference skills than their monolingual peers. It seems that the bilinguals in our sample were not hampered to use their text structure inference skills because of effortful word and sentence processing, nor did their text structure inference skills played a larger role for their text comprehension performances as one could expect from the compensation hypothesis.

It is noteworthy that monolinguals and bilinguals do not differ in their text structure inference skills, despite differences in knowledge of connectives and metacognitive knowledge, the two factors that have been put forward as pivotal to text structure inference skills. One explanation for these seemingly contradicting results could be that differences between monolinguals and bilinguals in knowledge of connectives and metacognitive knowledge are not large enough to result in differences in text structure inference skill. Compared to differences between monolinguals and bilinguals in general vocabulary knowledge, effect sizes for the difference in knowledge of connectives and metacognitive knowledge are quite small: around twice as small for knowledge of connectives (9 vs. 14%) and almost three times as small for metacognitive knowledge (5 vs. 14%). Another explanation might be that text structure inference skill, apart from knowledge of connectives and metacognitive knowledge, depends on other, language-independent skills, as for example reasoning skills or general intelligence, on which the bilinguals may outperform the monolinguals.

### Limitations and future directions

The present study had quite large attrition of test scores, therefore we consider replication of our study important to test the validity of our results. We also consider replication of our study important because our text structure inference skill test (although piloted thoroughly) is not used in any other study yet. In future studies it would also be interesting to explore whether there is a distinction between the productive part of the task (writing the summaries) and the receptive part of it (recognition of text structure, measured with multiple choice items). Furthermore, our correlational design prevents us from drawing conclusions about causality. We are therefore not able to clarify whether higher text structure inference skill leads to better text comprehension or vice versa. Moreover, in order to investigate whether knowledge of connectives and metacognitive knowledge are indeed crucial factors for inferring text structure, text structure inferences of readers during expository text reading have to be examined by readers who differ in knowledge of connectives and metacognitive knowledge levels.

Furthermore, we consider it important to stress that the robustness check we performed with a slightly larger sample size of 191 students indicated that the effect of text structure inference skill on expository text comprehension is not accounted for by knowledge of connectives and metacognitive knowledge for every reader. This result concurs with the assumption put forward in the previous paragraph that other language independent skills besides knowledge of connectives and metacognitive knowledge are important for text structure inference. On top of that, this result also indicates that for some readers there is a discrepancy between having the knowledge to infer text structure (i.e., knowledge of connectives and metacognitive knowledge) and applying this knowledge for text structure inference. Future research is therefore required to examine which other cognitions relate to students’ ability to infer text structure during reading.

A method that may be used to tap into online text structure inferences is measuring students’ reaction times to target words or sentences related to text structure (cf., Long, Oppy, & Seely, 1994; Lorch, Lorch, & Mogan, 1987; Ritchey, 2011). Collecting data about online text structure inferences could also be combined with intervention studies to examine whether readers who are trained specifically in knowledge of connectives and metacognitive knowledge are better in inferring text structure than peers who did not get specialized instruction in connectives, text structure and reading strategies to infer structure.

Both online and intervention studies will contribute to a better understanding of the conditions under which text structure inferences are made and which knowledge and skills are necessary to make these inferences. Such studies serve practical purposes as well: they may help teachers to improve students’ text structure inference skills and consequently their ability to create a coherent text representation.

### Implications for practice

The present study has shown a strong correlation between text structure inference skill and expository text comprehension. Although a correlation does not imply causality, many other studies have shown that training students on knowledge and skills necessary to infer text structure improves text comprehension (e.g., Cook & Mayer, 1988; Gordon, 1989; Meyer et al., 1989; Meyer & Poon, 2001; Moeken, Kuiken, & Welie, 2015; Paris et al., 1984; Wijekumar et al., 2013; Williams et al., 2004, 2009; Williams, 2005). Therefore we can safely assume that better text structure inference skill leads secondary school readers to better understanding of their expository texts. Because our study has shown that metacognitive knowledge and knowledge of connectives are key to infer text structure, we think that teaching these kinds of knowledge could be beneficial to secondary school students. A study of Welie et al., (2016) related to the present study also showed that knowledge of connectives and metacognitive knowledge are important for expository text comprehension: in this study an interaction between knowledge of connectives and metacognitive knowledge was found, which indicated that both types of knowledge had to be well developed in order to achieve a high level of expository text comprehension. Based on these results, it seems that combining the instruction of both kinds of knowledge could enhance the effect on reading comprehension skill. More specifically, besides knowing the meaning of connectives (knowledge of connectives), readers need to know the relevance of connectives and the appropriate reading strategies (metacognitive knowledge) to maximally benefit from them.

Also of interest to educational practitioners is the lack of interaction we found between text structure inference skill and other predictors (i.e., language background, reading proficiency level, reading fluency, vocabulary knowledge and knowledge of connectives). This finding seems to indicate that eighth graders with less than optimal cognitive resources (or a language minority background associated with one or more of these characteristics) draw similar benefits from better text structure inference skills. In other words, at secondary school, cognitive resources (fluency, vocabulary, etc.) do not seem to require a more developed level as a prerequisite to benefit from instruction on text structure inference skill. Secondary school teachers may therefore start to train students’ text structure inference skills regardless of these students’ cognitive development. This kind of instruction is necessary as many secondary school students seem to fail the desired level of expository text understanding (Hacquebord et al., 2004; Inspectie van het onderwijs, 2008; Kamil, 2003; Lemke et al., 2004; OECD, 2003, 2007; Perie et al., 2005; Welie, 2017), especially those students with a language minority background (for a review in North-American context, see August & Shanahan, 2006; for the Netherlands, see, for example Aarts & Verhoeven, 1999; Trapman et al., 2014; Van Gelderen et al., 2003).

## Footnotes

1 This method section has overlap with the method section of Welie, Schoonen, Kuiken, & Van den Bergh, (2016). Welie et al., (2016) used the same participants (and an additional 20 students) and the same tests (except for the text structure inference skill test) to examine the role of knowledge of connectives in expository text comprehension.

2 Adding text structure inference skill as a predictor in models that included sentence reading fluency and one or two of the following predictors as control variables led to the following results: knowledge of connectives (*χ*² (2) = 19.21, *p* = .00, Δr² = .08), metacognitive knowledge (*χ*² (2) = 20.27, *p* = .00, Δr² = .09), general vocabulary knowledge and metacognitive knowledge (*χ*² (2) = 19.21, *p* = .018.69, Δr² = .09), general vocabulary knowledge and knowledge of connectives *(χ*² (1) = 18.69, *p* = .00, Δr² = .06).
